# Repair of Orbital Post-Traumatic Wall Defects by Custom-Made TiNi Mesh Endografts

**DOI:** 10.3390/jfb10030027

**Published:** 2019-06-27

**Authors:** Valentin Shtin, Valeriy Novikov, Timofey Chekalkin, Victor Gunther, Ekaterina Marchenko, Evgeniy Choynzonov, Seung Baik Kang, Moon Jong Chang, Ji Hoon Kang, Aleksei Obrosov

**Affiliations:** 1 Tomsk Cancer Research Institute, Tomsk National Research Medical Center of the Russian Academy of Sciences, Tomsk 634028, Russia; 2 Research Institute of Medical Materials, Tomsk State University, Tomsk 634045, Russia; 3 Kang and Park Medical Co., R&D Center, Ochang 28119, Korea; 4 SMG-SNU Boramae Medical Center, Seoul National University, College of Medicine, Seoul 07061, Korea; 5 Department of Physical Metallurgy and Materials Technology, Brandenburg University of Technology, 03044 Cottbus, Germany

**Keywords:** diplopia, enophthalmos, orbital defect repair, TiNi mesh

## Abstract

Repairs of orbital post-traumatic and extensive malignant defects remain a major surgical challenge, in view of follow-up outcomes. Incorrect surgical management of injured facial structures results in cosmetic, ophthalmic, and social aftereffects. A custom-made knitted TiNi-based mesh (KTNM) endograft was employed to overcome post-surgical complications and post-resected lesions of the orbital area. Preoperative high-resolution computed tomography (CT) imaging and CAD modelling were used to design the customized KTNM in each case. Twenty-five patients underwent surgery utilizing the suggested technique, from 2014 to 2019. In all documented cases, resolution of the ophthalmic malfunction was noted in the early period. Follow-up observation evidenced no relapsed enophthalmos, hypoglobus, or diplopia as late complications. The findings emanating from our clinical observations allow us to claim that the KTNM indicated a high level of biocompatibility. It is simply modified intraoperatively to attach any desired shape/size for implantation and can also be screw-fixed, providing a good supporting ability. The KTNM precisely renders orbitozygomatic outlines and orbital floor, thus recovering the anatomical structure, and is regarded as an attractive alternative to Ti-based meshes and plates. Additionally, we report one of the studied cases, where good functional and cosmetic outcomes have been achieved.

## 1. Introduction

Deformities of the midface are caused by trauma, osteomyelitis, cystic lesions, neoplasia, and surgical treatment of tumours. Challenges in surgical management of orbital deformities faced in correcting such deformities are mainly due to the complicated anatomy and the multitude of functions that are affected by loss of tissue in this region. The facial skeleton may be arbitrarily divided into three different zones: Upper, middle, and lower third. The midface area formed by sixteen thin and compact bones consists of the sophisticated and interlinked constituents. Therefore, a lesion in even one of them simultaneously involves the adjacent structures. Most authors report that the surgical management of maxillary and zygomatic fractures can be quite challenging, with the potential for high rates of complications as evidenced in the literature [1,2].

Zygomatic-orbital complex injuries are the often-observed facial lesions resulting from traumas that have severe aesthetic and functional sequelae. Traumatic orbital injuries are rated as high, accounting for 23% of all midface fractures [3]. Displacement of orbital constituents leads not only to cosmetic defects but also to binocular malfunction (enophthalmos, persistent diplopia, etc.). Surgical treatment aimed at restoring the natural position of the eyeball is an obligatory procedure in almost all cases, even in the absence of muscle contraction in the fractured zone. As a rule, it interferes with the eye muscle strained, catalysing the displacement of the eyeball; thereby the iatrogenic aftereffect occurs, evidenced as aggravated eye motility, hypoglobus, and enophthalmos [4]. As such, the objective of revision surgery is to restore the integrity of the orbital cavity jeopardized due to complications or trauma, and to normalize the function of musculoskeletal structures. Unfortunately, this is hard to solve by a simple repositioning of the orbital walls and floor; therefore, further studies on feasibility with the aid of various implants are needed. Note that one of the adverse factors of midface reconstruction is highly virulent flora influencing the operating wound, which trigger the inflammation process in the implantation zone [5]. In addition, the subcranial region is an area of increased functional activity [6]. It is clear that an endograft, in which resilience to the adverse impacts along with the anisotropic compliance and versatility in terms of stress–strain is inherent, can therefore be the most advanced option. Such endograft is assumed to be designed individually to reproduce the required contours, allowing a surgeon to intraoperatively preshape it in situ for insertion through the smallest incision, shortening surgery time and improving cosmetic outcomes. The development of highly effective surgical techniques to repair the orbitozygomatic area with minimal risk of complications seems to be promising.

The desire to rehabilitate and to socialize the patient, giving them the opportunity to be a full member of society as soon as possible after severe injuries and extensive surgery, forces specialists to suggest novel decisions. That being the case, the global research community must focus on improving biochemical and biomechanical features, especially concerning implant surface interactions [7,8]. Almost all medical techniques on orbital plasty are aimed at applying various endografts and scaffolds made of inorganic materials, in order to minimize implant failures and to reduce the complication rates. Published reports are available on modern orbital defect repair, using alloys, polymers, ceramics, carbon composite compounds, etc. Each having different kinds of surface modification to alter the default host response [9,10].

In our view, there are some interrelated conditions, the fulfilment of which is mandatory for orbital endografts to be implanted. These conditions can be outlined as follows: (i) endograft’s microporous surface; (ii) superelastic behavior (up to 5% of reversible strain); (iii) overall thickness not exceeding 400 μm; (iv) customized design of the light mesh endograft. All mentioned specs turned out to have been resolved with the use of knitted TiNi-based mesh (KTNM) endograft.

In this article, a relatively easy reconstruction technique to fix the surgical faults, complications, and traumatic lesions of the orbital area using the KTNM is reported. Although our report addresses the revision surgical procedure, we are convinced that the technique can be successfully extended to the surgical treatment of fresh injuries to avoid possible complications manifesting as diplopia/enophthalmos and other deformities.

## 2. Case Report

The presented patient (K.) is a 32-year-old male, who is employed as a truck driver. He was admitted to the hospital of the Tomsk Cancer Research Institute with a complaint of vision errors, for the management of his facial injuries. According to him, in 2006 he had been injured on the job when his right maxilla was partially fractured, and he was then managed conservatively. In 2017 he suffered a traffic accident resulting in a completely fractured right maxilla and eyeball displacement. He received emergency care combined with eyeball surgical repositioning, followed by engrafting with the autogenous bone in a hospital. A month after his discharge, he noticed vision errors and was referred to our hospital to repair the right orbital wall in accordance with the suggested technique.

CT scans and MRI report (October 2017) revealed old fractures to the right maxilla, zygomatic bone, and showed uneven contours of the lower and medial walls of the right orbit, with fragments and tissues protruding medially and downwards. The preoperative ophthalmological findings are summarized and also collated with postoperative data in Table 1.

**Figure 1:**
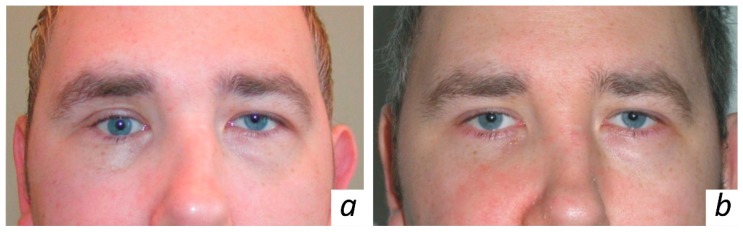
Frontal view of the patient K.: (**a**) preoperative asymmetric palpebral fissure, hypoglobus and enophthalmos; (**b**) follow up (six months after surgery) evident ocular symmetry with correction of hypoglobus and enophthalmos in his right eye.

**Figure 2:**
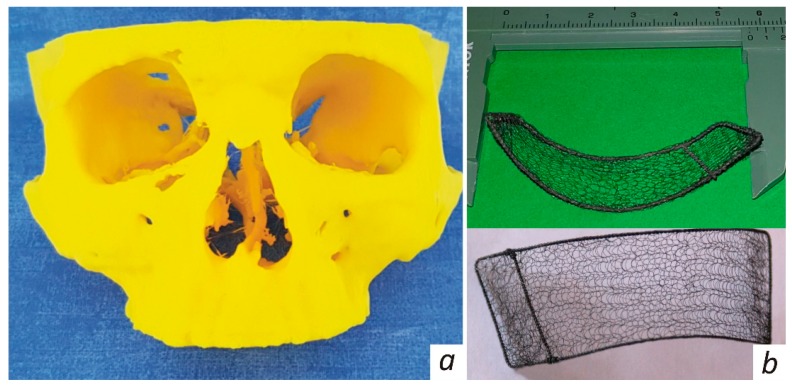
View of (**a**) printed 3D model and (**b**) as-fabricated endograft

The eye examination indicated diplopia of the right eye during outward and upward gaze as seen in Figure 1a. Thus, clinical examination revealed eyeball displacement resulting from the orbital fracture. The 3D model of the patient’s skull as indicated in Figure 2a was printed out using spiral CT scans.

Based on the 3D model, the customized KTNM was made as depicted in Figure 2b, then fitted, sterilized, and packed. The patient underwent endoscope-assisted reconstruction of the right orbital walls. The orbicularis oculi was exposed via subciliary incision along the lower orbital margin. Revision of the orbit was performed using a 4.0 mm telescope (in directions of view 0° and 30° wide angle) and endoscopic retractors. The defect of the lower and medial orbital walls containing orbital tissues protruding into the maxillary sinus was noted. Protruding orbital constituents were retrieved and repositioned. The next step was reconstruction of the medial and inferior orbital walls using the sterile KTNM. The frame of the latter was slightly predeformed and shrunk for smooth insertion, followed by deployment of the in situ KTNM, which was intraoperatively controlled using the C-arch. No additional fixation was applied to prevent KTNM migration, owing to its well-developed surface and inherent adhesiveness. The surgical wound was managed with primary intention in layers, taking care to avoid any tension across the stitch line.

**Figure 3:**
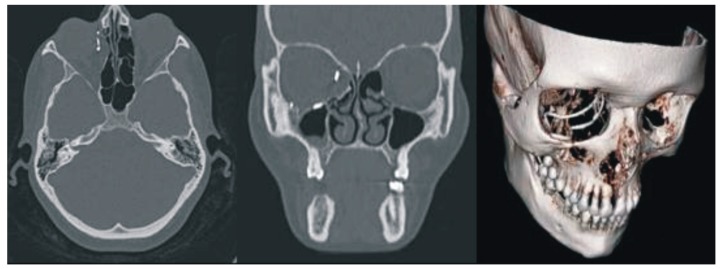
Facial CT images with 3D reconstruction at one month after surgery.

No major complications were observed in the postoperative period. Moderate retrobulbar edema was observed in the early postoperative period. The conjunctival fold up to 2 mm in thickness was seen on the bulbar conjunctiva at the 6 o’clock position. In the early postoperative term, binocular diplopia occurred in various positions of gaze. This might have been caused by edema and postoperative retrobulbar hematoma. A month later, clinical examinations and spiral CT scans with 3D reconstruction as depicted in Figure 3, indicated that the eyeball position in the repaired orbit symmetrically matched that of the intact side. The location of KTNM was the same as intraoperatively predetermined. The patient demonstrated the full range of movement of the eyes. There was no evidence of diplopia. Postoperative ophthalmologic findings are also given in Table 1, whereas the patient appearance six months after surgery is illustrated in Figure 1b.

Drawing on the data presented in Table 1 and the clinical observations obtained postoperatively, the anatomy of the right orbit, together with the right eyeball position, is argued to have been restored entirely, eliminating side effects.

**Figure 4:**
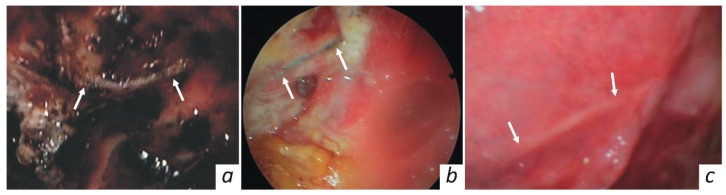
Endoscopic view of the repaired orbital area, taken at (**a**) 5, (**b**) 21, and (**c**) 35 days after surgery.

In the follow-up period, the reparative process in the treated orbital area was endoscopically monitored, as shown in Figure 4. On the fifth day the implantation zone was covered with fibrin. The implant site is seen to be not covered with tissues of the recipient area, whereas clots and granulation tissue are pointed out. On the twenty-first day, the wound surface seems to be completely cleared of necrotic masses and fibrin. Granulation tissue formation continued, and epithelialisation at the wound margin was noted. The KTNM was completely epithelialized within one month after surgery. No inflammatory changes were observed in the recipient zone.

After the patient spent five days in the hospital, he was discharged without any complaints and returned to his job two days later. He calls us every 4 months to confirm his well-being and health status.

## 3. Discussion

The features of surgical treatment in a case of post-traumatic or oncological midface defect management imply that it addresses the obvious functional and cosmetic concerns, including vision, breathing, phonation, chewing, and even digestion; by making such patients not feel inferior. Since the canons of reconstructive facial surgery were set in the last century, the feasibility of reconstructing the lesion and anatomy has now become a recognized fact [11,12]. According to those principles, the endograft must comply with the structure, consistency, shape, size, and function of the tissue to be repaired.

The current medical market offers various routes to circumvent the challenge faced by surgical society, and these can be classified as autografts, allografts, xenografts (embryonic, cadaveric, bovine tissues), inorganic implants (metals, polymers, etc.), as well as their combinations. For example, a well-known technique is using a portion of the temporalis to restore the orbital wall, in which the muscle is subsequently replaced by connective tissue, followed by the thick cicatricial tissue forming the orbital wall and floor [13,14]. Osteoplastic methods creating the rigid 3D architecture are supposed to resort to bone auto- and allografts which are frequently supplemented by prefabricated flaps on the vascular pedicle. The drawback of these techniques is their sophisticated surgical procedure, complicated by time consumption, sizable surgical wound, low contourability, donor site defect, prolonged epithelialisation, and lengthy healing. Moreover, from our own clinical experience, facial deformities caused by the resorption process were often noted and had to be critically assessed at a later period.

Silicone and polymer-based grafts are the most popular among inorganic biomaterials for midface repair. Besides, several studies have been actively pursued, which have been reported to evaluate the impact that combined metal-polymer biomaterials have on the host-vs-graft response and successful outcomes [15]. Evidence in the literature indicates that studies conducted on hydrogel grafts to restore the orbital walls are somewhat biased regarding prospects for a wide clinical practice [16]. Additionally, surgical composite grafts consisting of metal meshes embedded in the polyethylene matrix (pore size of 20–500 μm) are known as being currently explored [17]. All these techniques may ensure a satisfactory level of orbital configuration and patient tolerance in the short term. However, their being in permanent contact with the highly virulent flora coming from the paranasal sinuses and oral cavity does not allow us to choose any of the above-mentioned grafts as versatile. This imposes restrictions on the use of both polymer and hydrogel implants. Intraoperative contact with the flora may also adversely affect biodegradable and polymeric materials which can further sustain pathogenic agents and infectious processes. Biodegradable grafts are prone to foreign-body reaction, and have only fibrous connective tissue remaining after resorption. Therefore, increased complication rates related to the use of such allografts have been reported [18,19], wherein biodegradable grafts could only be used in small-scale orbital defects, and scars formed after implant resorption may influence functional outcomes. In this regard, studies focused on the search for metal-based grafts seem to be encouraging.

Among metallic biomaterials, porous titanium and Ti-based alloys, doped aluminium, iron, or niobium are known to be prominent in clinical deployment [20]. The Ti-based alloys are often the materials of choice, being of relatively low weight and density compared to stainless steels and Co-Cr alloys. Long-term in vivo studies of stainless steel or Co-Cr alloys have been reported to indicate an increased risk of development of cutaneous and systemic hypersensitivity reactions, whereas an excessive structural stiffness of these metallic materials may result in limited biointegration and stress shield-induced aftereffects [21]. The elastic modulus and stiffness of both stainless steel and Co-Cr alloys are also higher than that of Ti, which leads to greater stress shielding than in the case of Ti-based alloys. As such, the osseointegration level of stainless steel and Co-Cr is lower than that of Ti. With reference to [22], it means that the Ti-based grafts are encountered as engaging with the osseous tissue in direct contact, whereas the Co-Cr alloy can be a constituent of grafts that do not interact with the bone. Moreover, due to the surface oxide film, Ti-based alloys indicate a certain biocompatibility evident through a reduced graft-vs-host response and less abundant fibrous tissue. This has enabled the use of Ti-based implants as a good osteoplastic material throughout the body. Substantial shortcomings of the technique can however be observed from years of tracking, including the complexity of easy mounting of the stiff Ti graft [23,24], as well as the re-surgery that follows so as to correct or remove it in pediatric patients, which is a challenging exercise. Another crucial factor is inadequate corrosion resistance of the unwrought Ti surface. The rationale for such an assertion is explained with reference to titanium alloys subjected to alternating strain in the body, leading to the oxide film fissuring. Tissue fluids infiltrating into cracks may even cause crevice corrosion followed by implant failure, which has been observed in our clinical practice also. Currently, the deposition of corrosion-resistant coatings based on Ti(C,N) by a variety of methods is supposed to enhance the corrosion resistance of titanium alloys [25]. Another option is to modify the Ti surface by forming the superficial carbonitride gradient layers. However, such a provision undoubtedly leads to higher costs for commercially available Ti-based implants.

Whenever TiNi-based alloys are mentioned in the context of long-term implantable constructs, a combination of corrosion resistance and good biocompatibility with tissues is emphasized. When considering the benefits that make these alloys very attractive candidates for biomedical applications, it is also crucial to note the relative cheapness of unwrought semi-finished items (plaited frame and mesh as mentioned in our case) used for fabrication of the KTNM. The reviewed literature stresses the importance of customized design in modern reconstructive surgery, which is also relevant for the KTNM. The positive interaction between the KTNM and surrounding tissues was previously studied in animal models [7,26], and it was also assessed clinically in 120 cancer patients, as reported in Refs. [10,27], where the survivability of KTNM placed in highly virulent media was highlighted. The said studies indicated that the formation of bone tissue occurred through indirect osteogenesis: loose connective tissue followed by the formation of dense semi-formed connective tissue, which was replaced by fibrocartilage. In addition, histological examination revealed no signs of leukocyte infiltration or development of connective tissue on the KTNM’s periphery on day 14 after surgery. Loose connective tissue around the KTNM with no signs of inflammation was noted on day 28 after surgery, whereas apparent lymphocytic leukocyte infiltration around polypropylene mesh lasted up to 72 days [28]. Cell response to surface topography is a primary feature of the forming of many tissues. Surface roughness has a direct favorable influence on cellular morphology and proliferation. The filament that the KTNM is made from exhibits a micro-porous surface structure which reduces stress-shielding effect and encourages propitious tissue ingrowth. This means that intimate fusion of the mesh-tissue occurs, allowing fibroblasts to infiltrate the KTNM and form a new tissue across it.

A versatile ad hoc endograft for orbital repair continues to be a contentious issue. Possessing remarkable biocompatibility, demonstrating distensibility without impairment of mechanical properties in large defects or growing tissues, and mimicking the anisotropic compliance of the substituted tissue; it should be custom-made, easily fabricated, easily accessible, and affordable. It should require no special training or skills for surgical management complying with strict adherence to the rules of midface reconstructive treatment, and it is supposed to be combined with ordinary surgical techniques such as revising, screwing, stitching, etc. The larger the orbital lesion is, the more likely it is that a type of graft will be needed. If a graft is required to repair the orbit defect, the current medical market offers a certain quantity of grafts to choose from. However, it is often difficult to assign a customized design and physiological properties to mass-produced endografts. The customized KTNM is designed to interact for as long as it is in the body, in large deformations, showing comparable stress–strain behavior and negligible graft-vs.-host response, all of which favorably distinguish it from rivals.

## 4. Materials and Methods

Based on preoperative planning, the customized endograft is fabricated following the volume of upcoming repair as recommended in References [7,10,27]. Each KTNM is made of the TN-10 alloy superelastic filaments (60 µm in diameter), having a microporous, oxicarbonitride superficial layer [29,30]. The bio-mimic in vitro/vivo features of TiNi-based constructs have been reported to resemble the behavior of human body tissues [26,31]. The light mesh is double knitted by a process which interlinks each filament junction and provides for superelasticity in any direction, as indicated in [10]. Using the data personally acquired from CT scans and CAD modelling, the 3D model depicted in Figure 2a is printed out. Since such mesh enables us to get any desired shape or size without unravelling, it is then draped over the plaited frame made of the same alloy twisted wires (200 µm in diameter) according to the produced 3D model, aligned, and stitched. Finally, the KTNM, as seen in Figure 2b, which accurately reproduces the orbital contours to be repaired, is sterilized by gamma irradiation and kept in a sterile pack. Note that once the 3D model has been printed out the sterile KTNM would normally be dispatched the next day to a hospital.

Twenty-five patients (age ranging from 24 to 61 years) with post-traumatic facial defects and paranasal sinus tumors were treated according to the suggested technique, between 2014 and 2019. Most of them (15, 60%) were males previously treated after traffic accidents. Twenty-two patients underwent reconstruction of the lower and medial orbital walls, whereas three cases included repair of the lateral, lower, and medial orbital walls. The study was performed in accordance with the ethical principles outlined in the Declaration of Helsinki. The Ethical Committee of the Tomsk Cancer Research Institute approved the study protocol, with each patient providing their written consent before the upcoming intervention, for publication of the research findings.

Taking into account the cosmetic features, routine pre-, intra- and post-surgical procedures were performed using the endoscope set Karl Storz 40334101 Clearvision II. All patients were subjected to general anesthesia through a nasoendotracheal tube. The preseptal subciliary incision was made 2 mm caudal and parallel to the lower eyelash, then dissecting the orbicularis muscle 2 to 3 mm below the tarsal plate. In some complicated cases, mainly in cancer patients, after extensive resection the KTNM was screw-fixed to the infraorbital rim. The correct KTNM position was intraoperatively confirmed by obtaining CT scans using the C–arc.

The outcomes were assessed to confirm correction of the preoperative complaints, through clinical and outpatient observation for a period of one month using the following diagnostic algorithm:

(a) Ophthalmologic examination for errors in vision in all positions of gaze. The volume of eye movements, presence/absence of diplopia, hypophthalmos and enophthalmos were evaluated. The visual acuity and visual field were also studied. All diagnostic measures were performed before surgery, and at ten days, three weeks, and one month after reconstruction.
(b) Postoperative CT scanning. The examination was conducted in axial, coronal, and frontal projections using a spiral scanning program (1/1 mm sections, 1–1.5 pitch) with subsequent multiplanar reformations and 3D image. The image analysis was carried out in the modes of soft and bone tissue windows.
(c) Endoscopic examination including a tissue biopsy probe in the implantation zone.

## 5. Conclusions

The suggested simple surgical technique using custom-made KTNMs has been shown to be effective in the reconstruction of extensive and post-traumatic orbital lesions, taking up minimum time for the intervention and ensuring good cosmetic and functional outcomes without side effects. The customized KTNM is easily inserted and accurately replicates orbital contours, thus restoring the orbital architecture and correcting diplopia, hypoglobus, and enophthalmos. The position and vision function were recovered in all patients who received the KTNM. Thus, customised KTNMs exhibiting high adaptability strive to complement and enhance existing reconstruction approaches, by retaining the anatomy of the orbital cavity and correcting vision errors without postoperative aggravation. Additionally, they are economically justified with regard to surgery cost minimisation.

## Figures and Tables

**Table 1:** Pre- and postoperative data collected from the eye examination of patient K. (mm).

	Before Surgery		Ten Days after		One Month after	
	Right	Left	Right	Left	Right	Left
**Exophthalmometry**	22	18	19	18	18	18
**Hypophthalmometry**	−3	0	−1	0	0	0
**Vertical gauge**	25	23	24	23	23	23

## References

[1] Wolff J., Sándor G.K., Pyysalo M., Miettinen A., Koivumäki A.-V., Kainulainen V.T. (2013). Late Reconstruction of Orbital and Naso-orbital Deformities. Oral Maxillofac. Surg. Clin. N. Am..

[2] Mazock J.B., Schow S.R., Triplett R.G. (2004). Evaluation of ocular changes secondary to blowout fractures. J. Oral Maxillofac. Surg..

[3] Rajkumar G.C., Ashwin D.P., Singh R., Prashanth R., Rudresh K.B. (2015). Ocular Injuries Associated with Midface Fractures: A 5 Year Survey. J. Maxillofac. Oral Surg..

[4] Mustafa S., Evans P., Bocca A., Patton D., Sugar A., Baxter P. (2011). Customized titanium reconstruction of post-traumatic orbital wall defects: A review of 22 cases. Int. J. Oral Maxillofac. Surg..

[5] Park C., Marchiori E., Barber J., Cardon C. (2014). Orbital Fracture Leading to Severe Multifascial Space Infection Including the Parapharyngeal Space: A Report of a Case and Review of the Literature. Craniomaxillofacial Trauma Reconstr..

[6] Kim H.S., Jeong E.C. (2016). Orbital Floor Fracture. Arch. Craniofacial Surg..

[7] Gunther V., Radkevich A., Kang S.B., Chekalkin T., Marchenko E., Gunther S., Pulikov A., Sinuk I., Kaunietis S., Podgorniy V. (2019). Study of the knitted TiNi mesh graft in a rabbit cranioplasty model. Biomed. Phys. Eng. Express.

[8] Morais J.M., Papadimitrakopoulos F., Burgess D.J. (2010). Biomaterials/Tissue Interactions: Possible Solutions to Overcome Foreign Body Response. AAPS J..

[9] Bratton E.M., Durairaj V.D. (2011). Orbital implants for fracture repair. Curr. Opin. Ophthalmol..

[10] Muhamedov M., Kulbakin D., Gunther V., Choynzonov E., Chekalkin T., Hodorenko V. (2015). Sparing surgery with the use of TiNi-based endografts in larynx cancer patients. J. Surg. Oncol..

[11] Rohrich R.J. (1999). Maxillary Reconstruction: Functional and Aesthetic Considerations. Plast. Reconstr. Surg..

[12] Wells M.D., Luce E.A. (1995). Reconstruction of midfacial defects after surgical resection of malignancies. Clin. Plast. Surg..

[13] Krzymański G., Dabrowski J., Przybysz J., Domański W., Biernacka B., Piętka T. (2012). Temporal muscle flap in reconstruction of maxillo-facial tissues. Współczesna Onkol..

[14] Uyar Y., Kumral T.L., Yıldırım G., Kuzdere M., Arbag H., Jorayev C., Kılıç M.V., Gümrükçü S.S., Yildirim G., Kilic M.V. (2015). Reconstruction of the Orbit With a Temporalis Muscle Flap After Orbital Exenteration. Clin. Exp. Otorhinolaryngol..

[15] Neumann A., Kevenhoerster K. (2009). Biomaterials for craniofacial reconstruction. GMS Curr. Top. Otorhinolaryngol. Head Neck Surg..

[16] Kim E.L., Bernardino C.R., Levin F. (2016). Orbital volume augmentation using expandable hydrogel implants in acquired anophthalmia and phthisis bulbi. Orbit.

[17] Kim S.W., Han H.H., Oh D.Y., Moon S.H., Lee J.H., Rhie J.W., Ahn S.T. (2012). Orbital Roof Reconstruction Using Porous Polyethylene Sheet With Embedded Titanium. J. Craniofacial Surg..

[18] Villarreal P.M., Monje F., Morillo A.J., Junquera L.M., González C., Barbón J.J. (2002). Porous Polyethylene Implants in Orbital Floor Reconstruction. Plast. Reconstr. Surg..

[19] Baumann A., Burggasser G., Gauss N., Ewers R. (2002). Orbital floor reconstruction with an alloplastic resorbable polydioxanone sheet. Int. J. Oral Maxillofac. Surg..

[20] Brunette D.M., Tengvall P., Textor M., Thomsen P. (2001). Titanium in Medicine.

[21] Ivanova E.P., Bazaka K., Crawford R.J. (2014). Metallic biomaterials: types and advanced applications. New Functional Biomaterials for Medicine and Healthcare.

[22] Prasad K., Bazaka O., Chua M., Rochford M., Fedrick L., Spoor J., Symes R., Tieppo M., Collins C., Cao A. (2017). Metallic Biomaterials: Current Challenges and Opportunities. Materials.

[23] Degala S., Shetty S.K., Biddappa L. (2012). Reconstruction of Post-traumatic Internal Orbital Wall Defects with Titanium Mesh. J. Maxillofac. Oral Surg..

[24] Vignesh U., Mehrotra D., Dichen, Anand V., Howlader D. (2017). Three dimensional reconstruction of late post traumatic orbital wall defects by customized implants using CAD-CAM, 3D stereolithographic models: A case report. J. Oral Boil. Craniofacial Res..

[25] Kirmanidou Y., Sidira M., Drosou M.-E., Bennani V., Bakopoulou A., Tsouknidas A., Michailidis N., Michalakis K. (2016). New Ti-Alloys and Surface Modifications to Improve the Mechanical Properties and the Biological Response to Orthopedic and Dental Implants: A Review. BioMed. Res. Int..

[26] Zaworonkow D., Chekan M., Kusnierz K., Lekstan A., Grajoszek A., Lekston Z., Lange D., Chekalkin T., Kang J.-H., Gunther V. (2018). Evaluation of TiNi-based wire mesh implant for abdominal wall defect management. Biomed. Phys. Eng. Express.

[27] Kulbakin D., Chekalkin T., Muhamedov M., Choynzonov E., Kang J.-H., Kang S.-B., Gunther V. (2016). Sparing Surgery for the Successful Treatment of Thyroid Papillary Carcinoma Invading the Trachea: A Case Report. Case Rep. Oncol..

[28] Kelly M., MacDougall K., Olabisi O., McGuire N. (2016). In vivo response to polypropylene following implantation in animal models: A review of biocompatibility. Int. Urogynecology J..

[29] Anikeev S., Hodorenko V., Gunther V., Chekalkin T., Kang J.-H., Kang S.-B. (2018). The effect of mechano-chemical treatment on structural properties of the drawn TiNi-based alloy wire. Mater. Res. Express.

[30] Gunther S., Chekalkin T., Hodorenko V., Kang J.H., Kim J.S., Gunther V. (2018). Impact of infrared radiation on oxide layer of ultrathin TiNi-based alloy wire. Adv. Mater. Lett..

[31] Kokorev O., Hodorenko V., Chekalkin T., Gunther V., Kang S.-B., Chang M.-J., Kang J.-H., Kang S.-B. (2019). Evaluation of allogenic hepato-tissue engineered in porous TiNi-based scaffolds for liver regeneration in a CCl4-induced cirrhosis rat model. Biomed. Phys. Eng. Express.

